# In‐field detection and characterization of B/Victoria lineage deletion variant viruses causing early influenza activity and an outbreak in Louisiana, 2019

**DOI:** 10.1111/irv.13246

**Published:** 2024-01-05

**Authors:** Bo Shu, Malania M. Wilson, Matthew W. Keller, Ha Tran, Theresa Sokol, Grace Lee, Benjamin L. Rambo‐Martin, Marie K. Kirby, Norman Hassell, Danielle Haydel, Julie Hand, David E. Wentworth, John R. Barnes

**Affiliations:** ^1^ Virology, Surveillance and Diagnosis Branch, Influenza Division Centers for Disease Control and Prevention Atlanta Georgia USA; ^2^ Louisiana Department of Health Office of Public Health Laboratory Baton Rouge Louisiana USA; ^3^ Louisiana Department of Health Office of Public Health, Infectious Disease Epidemiology New Orleans Louisiana USA

**Keywords:** amino acid deletion, B/Victoria lineage, in‐field detection, influenza outbreak

## Abstract

**Background:**

In 2019, the Louisiana Department of Health reported an early influenza B/Victoria (B/VIC) virus outbreak.

**Method:**

As it was an atypically large outbreak, we deployed to Louisiana to investigate it using genomics and a triplex real‐time RT‐PCR assay to detect three antigenically distinct B/VIC lineage variant viruses.

**Results:**

The investigation indicated that B/VIC V1A.3 subclade, containing a three amino acid deletion in the hemagglutinin and known to be antigenically distinct to the B/Colorado/06/2017 vaccine virus, was the most prevalent circulating virus within the specimens evaluated (86/88 in real‐time RT‐PCR).

**Conclusion:**

This work underscores the value of portable platforms for rapid, onsite pathogen characterization.

## 1 BACKGROUND

During the 2016–2017 influenza season, the influenza B (Inf B/Victoria (B/VIC) lineage variant viruses emerged with two or three amino acid (AA) deletions within the K_162_N_163_D_164_ region of the hemagglutinin (HA) protein and rapidly spread worldwide.^1^ These deletion variant viruses are antigenically distinct from each other and from the progenitor B/VIC virus that lacked the deletions, resulting in four co‐circulating subclades that were genetically and antigenically distinct during the timeframe of this outbreak: V1A (no deletion), V1A.1 (two‐AA deletion [2DEL]), and V1A.2 and V1A.3 (three‐AA deletion [3DEL]).^2^

To distinguish the B/VIC subclades, we developed a B/VIC lineage deletion detection triplex real‐time RT‐PCR (VIC DEL triplex rRT‐PCR) assay that used one set of forward and reverse primers and three deletion‐specific probes to detect B/VIC V1A (no deletion), V1A.1 (2DEL), and V1A.2/V1A.3 (3DEL) subclade viruses.^3^ The VIC DEL triplex rRT‐PCR allowed us to utilize PCR as a surrogate for antigenicity by distinguishing circulation of antigenic variants of B/VIC during the epidemic season and determining if circulating B/VIC viruses were similar to V1A.1 (2DEL) vaccine virus, B/Colorado/06/2017.

In August 2019 in Louisiana, the proportion of healthcare visits for influenza‐like illness (ILI) began to increase, primarily in children, and presented earlier than previous seasons.^4^ One large pediatric healthcare facility in New Orleans (Facility A) reported 1268 B/VIC virus infections confirmed by the Louisiana Department of Health (LDH) with the CDC Human Influenza Virus Real‐Time RT‐PCR Diagnostic Panel Influenza B Lineage Genotyping Kit,^5^ including 23 hospitalizations from July 31 to November 21, 2019. During this period, LDH reported one pediatric death associated with B/VIC virus infection.^4^

Due to the uncharacteristically large outbreak, rapid viral characterization was needed to determine if this was a known circulating virus or a new subclade. During November 2019, Louisiana declared a state of emergency after a cybersecurity attack on state government servers limited the connectivity of the LDH and their ability to perform genetic characterization. Our team deployed to the LDH with our mobile influenza analysis (*Mia*) next‐generation sequencing platform^6^ and our portable B/VIC DEL triplex rRT‐PCR assay, both of which were not reliant on the LDH system, to determine the viral subclades.

## 2 MATERIALS AND METHODS

### 2.1 Clinical specimens and in‐field RNA extraction

Sixty‐five respiratory specimens submitted to the LDH and 23 specimens from patients hospitalized in Louisiana with InfB virus infection between July 31 and November 21, 2019, were included in the study. These 88 specimens were previously determined to be InfB and B/VIC positive via testing by the LDH with the CDC Influenza B Lineage Genotyping Kit^5^ (Table S1). RNA from these 88 specimens was manually extracted onsite at the LDH using Akonni TruTip nucleic acid purification kit according to the previously established *Mia* protocol.^6^

### 2.2 VIC lineage deletion detection triplex real‐time RT‐PCR assay

The VIC DEL triplex rRT‐PCR assay includes a single set of conserved amplification primers and three deletion‐specific dual‐labeled hydrolysis probes, including VIC 2_Del, Vic 3_Del, and Vic No_Del probes. Three probes, targeted on the deletion region of the HA gene of B/VIC viruses, were designed to specifically detect and differentiate B/VIC V1A.1, V1A.2/V1A.3, and the V1A genetic subclade (no deletion) (V1A‐NoDel) viruses (Table S1 and Figure S1). The VIC DEL triplex rRT‐PCR probe's fluorescence and quenchers labeling were described previously^3^ (Table S1).

### 2.3 In‐field detection of influenza B/Victoria lineage deletion variant viruses

The VIC DEL triplex rRT‐PCR assay was performed in‐field using a portable, 4.5 lb real‐time PCR instrument, the Quantabio “Q” portable qRT‐PCR instrument (Beverly, MA, USA) that tests 48 samples in ~100 min/run. The rRT‐qPCR reactions setup and thermocycling conditions were previously described.^3^

### 2.4 Onsite and follow‐up sequencing

Sequencing was performed in‐field as described previously^6^ with some modifications. Here, InfB specific primers were used to amplify the RNA via SuperScript IV one‐step RT‐PCR. Raw fast5 read files were base called and demultiplexed with Guppy v.2.3.7 using default parameters on a MinIT GPU device (Oxford Nanopore Technologies, Oxford, UK). Reads were mapped and assembled into influenza genomes using IRMA v.0.6.7 with a MinION configuration module.^7^ Plurality consensus sequences for each segment were used in analyses. Samples were subsequently transferred to CDC in Atlanta where the RNA was re‐amplified using SuperScript III based multisegment RT‐PCR (MRT‐PCR) followed by Illumina (Illumina, San Diego, CA, USA) sequencing and analysis by the Influenza Division's surveillance pipeline.^7^

The AA deletion and substitutions of V1A HA sequences were analyzed using the BioEdit program (http://www.mbio.ncsu.edu/bioedit/). Phylogenetic analysis was performed using Molecular Evolutionary Genetics Analysis software (version 7.0 http://megasoftware.net). The evolutionary history was inferred using the maximum likelihood method. The evolutionary distances were computed using the Tamura–Nei method.^8^

Sequences obtained from this study have been submitted to the Global Initiative on Sharing All Influenza Data (GISAID) at https://giasiad.org/, and the accession numbers are listed in Table S2.

## 3 RESULTS

### 3.1 Performance of VIC deletion detection triplex rRT‐PCR assay in‐field

Eighty‐eight RNAs extracted from clinical specimens positive for InfB and B/VIC targets by the CDC Influenza B Lineage Genotyping Kit were tested in‐field using the VIC DEL triplex rRT‐PCR assay on a portable “Q” qPCR instrument. Eighty‐six samples tested positive with the VIC 3_Del probe, one sample was positive with the VIC 2_Del probe, and one sample was negative for both the VIC 3_Del probe and VIC 2_Del probe. All 88 samples tested negative for the VIC No_Del probe. The Ct values of viruses positive for the VIC DEL triplex rRT‐PCR assay were comparable to the Ct value of InfB (Tables 1 and S1).

**TABLE 1:** In‐field performance of B/VIC DEL triplex rRT‐PCR assay.

		B/VIC virus subclade^a^		Total
		V1A.1	V1A.3	
VIC DEL triplex rRT‐PCR assay	VIC 2_Del	1	0	1
	VIC 3_Del	0	86	86
	Negative^b^	0	1	1
Total		1	87	88

^a^ B/VIC V1A genetic subclades were determined from HA top Blast hit for the consensus sequence at 20× average coverage.

^b^ All three targets of B/VIC DEL triplex rRT‐PCR, including VIC 2_Del, VIC 3_Del, and VIC No_Del were tested negative.

### 3.2 Onsite and follow‐up sequencing results

We attempted onsite sequencing for 72 specimens using the *Mia* pipeline.^8^ Of these, 22 samples produced HA sequences with at least 20× coverage. Onsite alignments of those sequences confirmed the rRT‐PCR results.

**FIGURE 1:**
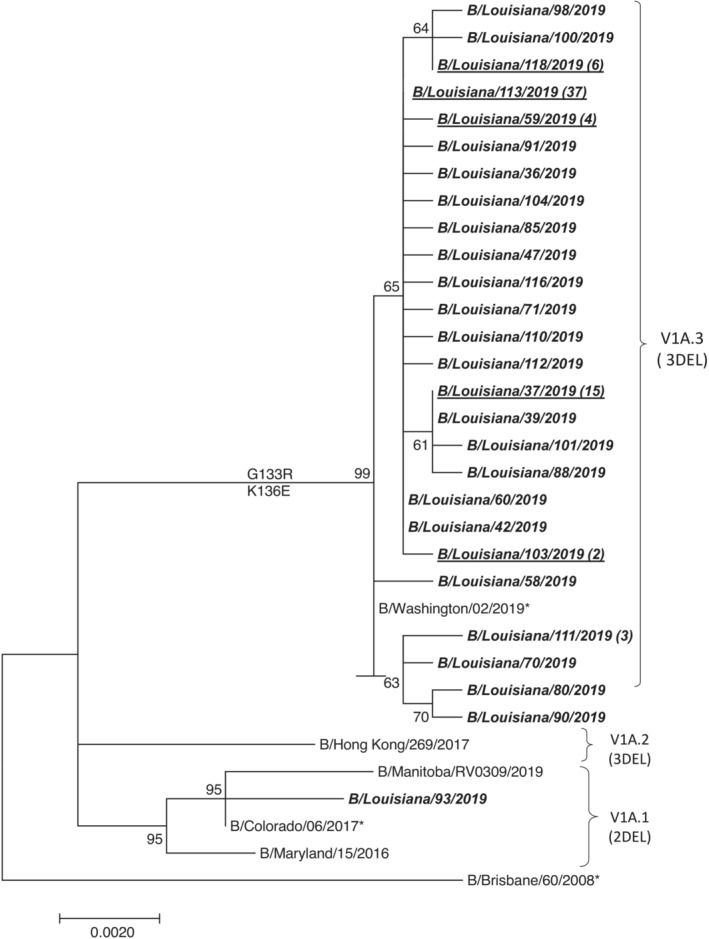
Evolutionary relationship of hemagglutinin gene among influenza B/Victoria V1A viruses. HA (coding sequence) phylogenetic tree with representative viruses of three subclades of V1A amino acid deletion viruses V1A.1 (2DEL), V1A.2 (3DEL), and V1A.3 (3DEL) indicated by the bars on the right. The quantityr and representative viruses from all identical HA sequences obtained from this study are shown in bold and italic face. The value in the parentheses indicates the quantity of identical sequences. The influenza B vaccine viruses are indicated with *. The bootstrap value (1000 replicates) of HA gene is shown next to the branches.

Through follow‐up MRT‐PCR amplification and Illumina sequencing, we obtained full InfB genomes for 88 specimens. The HA sequence from one VIC 2_Del positive specimen by rRT‐PCR was 2DEL and fell into the V1A.1 subclade in the phylogenetic tree (A/Louisiana/93/2019). Based on sequencing results of this study, the sensitivity and specificity of this triplex rRT‐PCR assay is 98.86% (87/88) and 100%, respectively. Eighty‐six specimens were VIC 3_Del positive, and the one negative specimen by rRT‐PCR was 3DEL (B/Louisiana/37/2019). All 3DEL confirmed samples fell into the V1A.3 subclade represented by the B/Washington/02/2019 virus. Among these 87 V1A.3 subclade viruses, 67 viruses had six unique HA genes. Eighty‐seven V1A.3 subclade viruses, together with B/Washington/02/2019, contained the HA1 G133R and K136E AA substitutions (Table 1; Figures 1 and S2).

## 4 DISCUSSION

This in‐field testing result demonstrated that the VIC DEL triplex rRT‐PCR assay was consistent with the CDC Influenza B Lineage Genotyping Kit. Eighty‐seven of the 88 specimens tested positive for either the VIC 2_Del or VIC 3_Del probes. The one specimen that was negative for the multiplex but confirmed as 3DEL via sequencing may have had a lower viral load since the Ct value for InfB was >30. It is also possible that the sample was inaccurately pipetted during the in‐field work, leading to a negative result on this specimen for the VIC DEL triplex. The rRT‐PCR result for a subset of samples was further confirmed by onsite and follow‐up sequencing.

Since the 2009–2010 influenza season, the B/Brisbane/60/2008 (V1A) has been the B/VIC vaccine virus strain for 8 years, indicating limited antigenic drift across these influenza seasons. Nevertheless, InfB virus emerged unusually early in the 2019–2020 influenza season and accounted for 59% of influenza positives reported from September 29 to December 14, 2019 by public health laboratories. Ninety‐seven percent of the InfB positive viruses tested for lineage belonged to the B/VIC lineage.^9^

In our investigation, all but one virus isolated in the field belonged to the V1A.3 subclade. The sequences of these V1A.3 subclade viruses were similar to B/Washington/02/2019, which was named as the vaccine strain for the B/VIC component of the influenza vaccine in the 2020 influenza season,^2^, ^10^ and contained 3DEL and HA1 G133R and K136E AA substitutions. These changes distinguished them from the vaccine virus at the time, B/Colorado/06/2017. Only one virus sequenced in the investigation was classified as a V1A.1 subclade, clustering with B/Colorado/16/2017. According to our findings, during this period, the predominantly circulating InfB viruses were B/VIC V1A.3 subclade, which began circulating in the United States in the latter half of the 2018–19 influenza season.^11^ Since then, B/VIC V1A.3 subclade has been the predominantly circulating virus strain in the United States.^12^, ^13^ The antigenic distance between the circulating viruses and the vaccine component may have contributed to this unexpectedly large and early outbreak of B/VIC. However, Owusu et al. showed that patients vaccinated with a B/VIC V1A.1 virus cross reacted well with B/VIC viruses containing the 3DEL, so the current vaccine should have provided at least some protection against circulating B/VIC V1A.3 viruses.^4^ Nonetheless, early outbreak investigations such as this allow for updated public health guidance if viruses differ antigenically from recommended vaccines.

## AUTHOR CONTRIBUTIONS

**Bo Shu**: In‐field real‐time RT‐PCR testing; writing—original draft. **Malania Wilson and Matthew Keller**: In‐field and follow‐up sequencing. **Ha Tran and Danielle Haydel**: Specimen testing; influenza B virus genotyping. **Theresa Sokol, Grace Lee and Julie Hand:** Epidemiologic investigation of the outbreak. **Benjamin Rambo‐Martin and Norman Hassell**: Sequence analysis. **Marie Kirby**: Writing—original draft; writing—reviewing and editing. **David Wentworth and John Barnes**: Supervision; writing—reviewing and editing.

## CONFLICT OF INTEREST STATEMENT

None declared.

### PEER REVIEW

The peer review history for this article is available at https://www.webofscience.com/api/gateway/wos/peer-review/10.1111/irv.13246.

## Supporting information


**Table S1.** CDC Influenza B/Victoria Deletion triplex real‐time RT‐PCR assay primer probe sequences.
**Table S2.** In‐field performance of B/VIC deletion detection triplex real‐time RT‐PCR assay with clinical specimens in comparison with InfB assay of the CDC Flu rRT‐PCR Dx Panel Influenza B Lineage Genotyping Kit.
**Figure S1.** Representative V1A viruses within B/VIC deletion detection triplex real‐time RT‐PCR primer and probe region.
**Figure S2.** Sequence logo of HA protein diversity of influenza B/Victoria V1A viruses isolated in‐field in Louisiana.

## Data Availability

Sequences obtained from this study have been submitted to the GISAID at https://giasiad.org, and the accession numbers are listed in Table S2. All additional associated data is shared in the Supporting Information for the manuscript.
