# Supplementary material for: In‐field detection and characterization of B/Victoria lineage deletion variant viruses causing early influenza activity and an outbreak in Louisiana, 2019
Source: Influenza Other Respir Viruses. 2024 Jan 5;18(1):e13246. doi: 10.1111/irv.13246 (PMC10767671; doi:10.1111/irv.13246)
Supplement: Supplementary file 1 — Table S1. CDC Influenza B/Victoria Deletion triplex real‐time RT‐PCR assay primer probe sequences. Table S2. In‐field performance of B/VIC deletion detection triplex real‐time RT‐PCR assay with clinical specimens in comparison with InfB assay of the CDC Flu rRT‐PCR Dx Panel Influenza B Lineage Genotyping Kit. Figure S1. Representative V1A viruses within B/VIC deletion detection triplex real‐time RT‐PCR primer and probe region. Figure S2. Sequence logo of HA protein diversity of influenza B/Victoria V1A viruses isolated in‐field in Louisiana. [file IRV-18-e13246-s001.docx]

**TABLE S1** CDC Influenza B/Victoria Deletion triplex real-time RT-PCR assay primer probe sequences

^a^Nucleotide Positions are according to HA gene of B/Brisbane/60/2008(GenBank accession no CY073893)

^b^The BHQ and LNA probe has 6-carboxyfluorescein (FAM) and BHQ™ 1 at the 3’ end

^c^A triplet of Locked Nucleotide Acid residues

^d^The BHQPlus Probe has hexachlorofluorescein (Hex) at 5' end and BHQ™1 at 3' end

^e^The BHQPlus Probe has CAL Fluor Red 610 at 5' end and BHQ™2 at 3' end

The BHQPlus probe employs C-5 propynyl-dC (pdC) for dC and C-5 propynyl-dU (pdU) for dT substitutions (Glen Research Corporation, Virginia, U.S)

**TABLE S2** In-field performance of B/VIC deletion detection triplex real-time RT-PCR assay with clinical specimens in comparison with InfB assay of the CDC Flu rRT-PCR Dx Panel Influenza B Lineage Genotyping Kit

The HA sequences were performed in-field and follow-up sequencing from specimens, and sequence accession numbers were signed

The HA sequences from specimens 2-37, 39-52, 54-58, 60-62 and 64-65 are identical to B/Louisiana/113/2019 (specimen 1), B/Louisiana/69/2019 (specimen 38), B/Louisiana/118/2019(specimen 53), B/Louisiana/59/2019 (specimen 59) and B/Louisiana/111/2019 (specimen 63), respectively. while HA sequences from specimens 66 and 67 are identical. The identical sequences were underlined.

**FIGURE S1** Representative V1A viruses within B/VIC deletion detection triplex real-time RT-PCR primer and probe region

One V1A.1 and the representative V1A.3 viruses from the in-field study in comparison to V1A subclade reference virus strains. The Conserved primers and B/VIC deletion specific probes were designed to specifically detect and differentiate influenza B Victoria lineage deletion variant viruses.

500 510 530 540 550 580 590

...|....|....|....|... ...|....|....|....|....|....| ....|....|....|....|....|.

**B/Brisbane/60/2008_V1A**  **GATTTTTCGCAACAATGGCTTG~~CGTCCCAAAAAACGACAAAAACAAAACAG~~GAAGTACCATACATTTGTACAGAAGG**

**VIC No_Del Probe**  **.................**

**B/Colorado/06/2017_V1A.1**  **....C................. .......------................ ............G.............**

**B/Louisiana/93/2019_V1A.1 ....C................. .......------................ ............G.............**

**VIC 2_Del Probe**  **.......------........**

**B/Washington/02/2019_V1A.3** **....C................. .......---------............. ..........................**

**B/Louisiana/113/2019_V1A.3 ....C................. .......---------............. ..........................**

**VIC 3_Del Probe**  **.....---------............**

**Forward and reverse primer** **....Y..Y..... ......... ----------------------------- ............R.............**

**FIGURE S2** Sequence logo of HA protein diversity of influenza B/Victoria V1A viruses isolated in-field in Louisiana


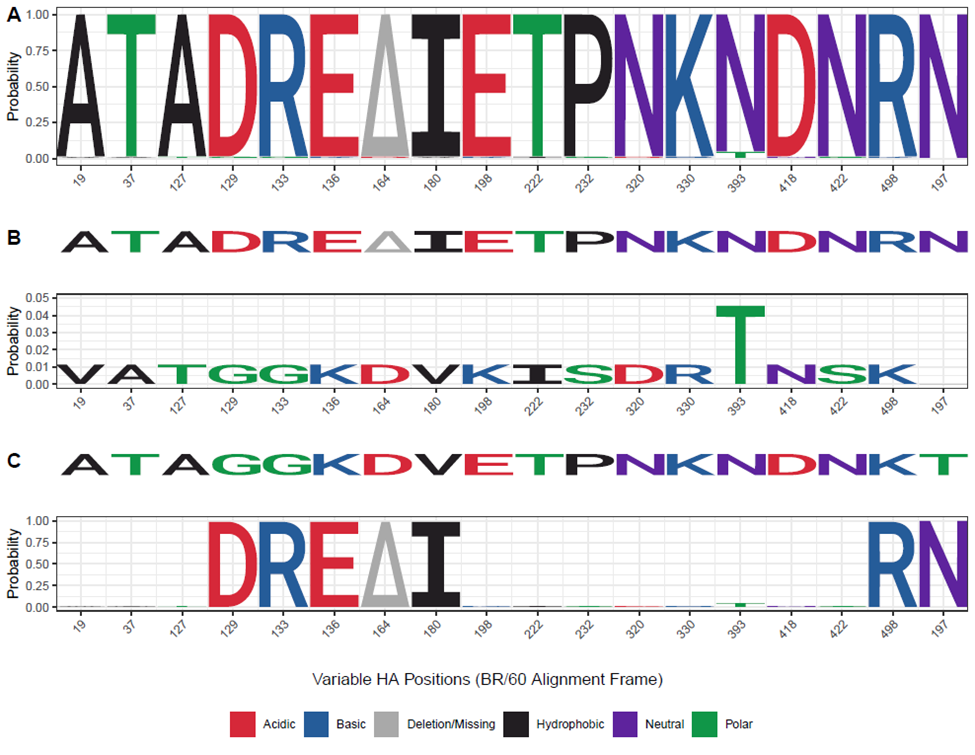


Amino acid positions are according to HA protein of B/Brisbane/60/2008(BR/60, GenBank accession no CY073893).

A. Sequence logo of protein diversity of positions variable to either A/Washington/02/2019 or A/Colorado/06/2017.

B. Sequence logo showing only variable differences to A/Washington/02/2019, with A/Washington/02/2019 sequence at the top of the logo for reference.

C. Sequence logo showing only variable differences to A/Colorado/06/2017, with A/Colorado/06/2017 sequence at the top of the logo for reference.
